# Supplementary material for: Complex‐centric proteome profiling by SEC‐SWATH‐MS
Source: Mol Syst Biol. 2019 Jan 14;15(1):e8438. doi: 10.15252/msb.20188438 (PMC6346213; doi:10.15252/msb.20188438)
Supplement: Supplementary file 6 — Dataset EV5 [file MSB-15-e8438-s006.zip › feature_plots_corum/2598;281.pdf]

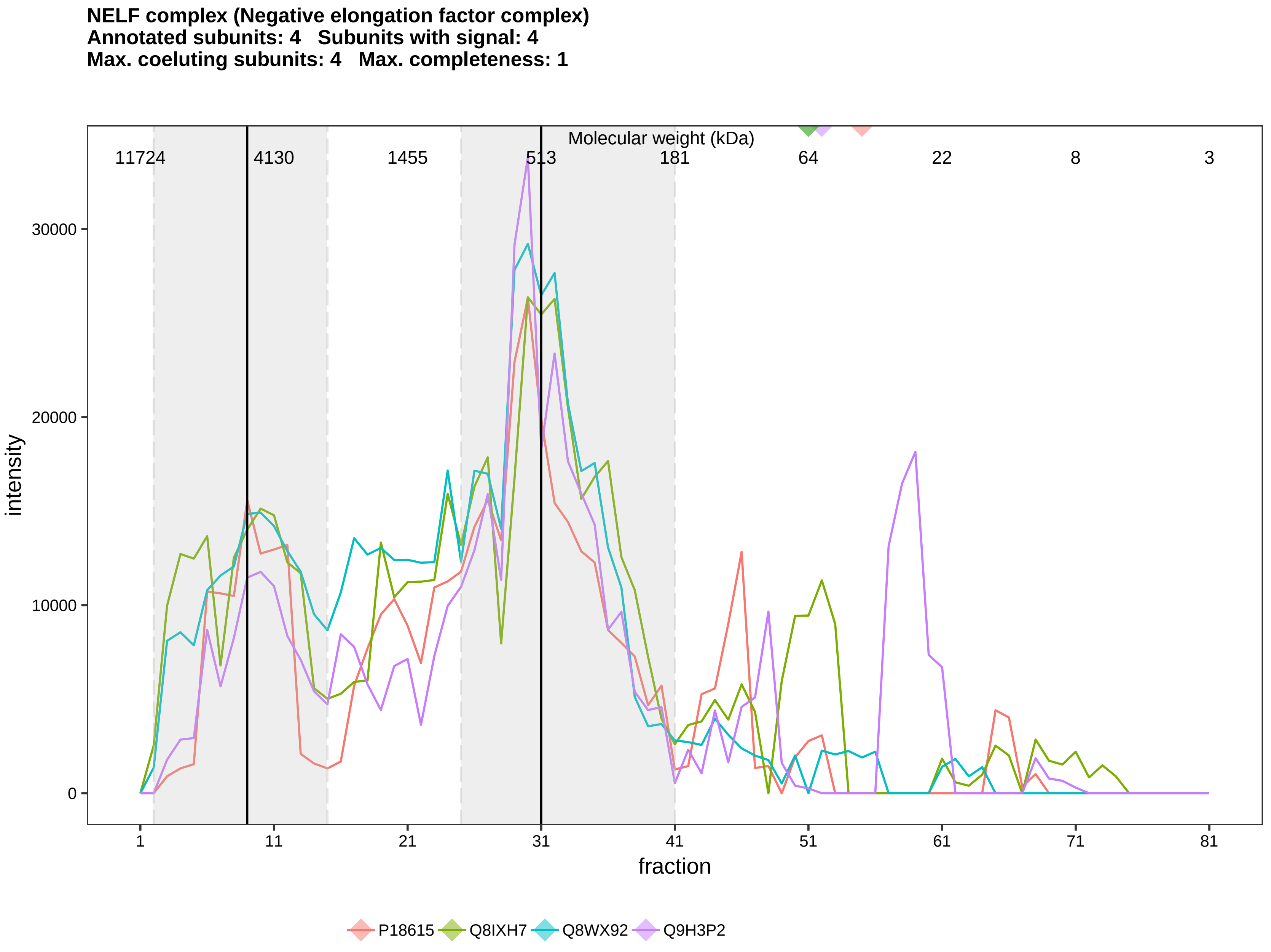

NELF complex (Negative elongation factor complex)
Annotated subunits: 4   Subunits with signal: 4
Max. coeluting subunits: 4   Max. completeness: 1
Molecular weight (kDa)
11724
4130
1455
513
181
64
22
8
3
intensity
30000
20000
10000
0
1
11
21
31
41
51
61
71
81
fraction
P18615
Q8IXH7
Q8WX92
Q9H3P2
